# Research on the Construction of a Nursing Education Management Model Based on a Small Data-Driven Model and Its Application

**DOI:** 10.1155/2022/3099794

**Published:** 2022-03-24

**Authors:** Qingna Lv, Yanyun Zhang, Yanyan Li, Yang Yu

**Affiliations:** ^1^Nursing Department, The Second Affiliated Hospital of Dalian Medical University, Dalian, Liaoning 116023, China; ^2^College of Nursing, Inner Mongolia Minzu University, Tongliao, Inner Mongolia Autonomous Region 028000, China; ^3^College of Nursing, Chongqing Medical and Pharmaceutical College, Shapingba, Chongqing 401331, China

## Abstract

Based on the concept of responsible holistic nursing care, a whole-process dual-tutor nursing practice model is established and its application effects are explored. This paper firstly reviews the research progress of nursing workload prediction methods at home and abroad, in order to provide a reference for clinical nursing workers in China to choose a scientific, reasonable, and easy-to-use nursing workload prediction method. It is proposed to construct a nursing education management model based on small data to provide ideas and references for nursing education management to effectively predict the evolutionary trend of students' behaviour and improve the level of accurate services. The experimental group adopted a dual-tutor responsibility system for the whole-process nursing practice model, including a complete three-level supervision system: a dual-tutor teaching system, a PDCA responsibility system for continuous improvement, and a multichannel teacher-student interaction platform; the control group adopted the traditional nursing practice model.

## 1. Introduction

In recent years, with the acceleration of the internationalization of medical education and the localization of international standards, nursing education has taken on the multiple missions of optimizing the cultivation of talents, leading to the development of disciplines and deepening the connotation of the profession, and the development of education needs to adapt to the development of social economy, the progress of science and technology, and the coordination of health undertakings [1]. The former Ministry of Health and the Ministry of Education jointly put forward the strategic point of building an integrated teaching mode of industry-university-research and clearly defined the development goal of deepening teaching reform and cultivating higher technical application talents [2]. The former Ministry of Health and the Ministry of Education jointly put forward the strategic point of building an integrated teaching model of industry-academia-research, specifying the development goal of deepening teaching reform and cultivating higher technical application-oriented talents. The changes in the clinical environment have put clinical nursing education in a new period of conceptual transformation and practical reform, and it is one of the core issues of nursing higher education to realize an effective interface and buffer between theory and practice and to explore a clinical practice model that fits the cultivation objectives of higher nursing education at this stage [3].

The information system-based nursing workload forecasting method refers to the use of information system retrieval and statistical functions to present medical order entries and nursing project data that reasonably and effectively reflect nursing workload so as to analyse and forecast nursing workload [4]. Currently commonly used in the USA is the GRASP nursing management information system (Grace Reynolds Application and Study of Peto, GRASP), which predicts workload and manpower in advance by forming a database of nursing workload for different categories of patients, depending on the type of patient admitted each day [5].

The GRASP system divides nursing items into 11 categories with 50 nursing operations and assigns a fixed number of points to each operation based on the average time spent on each item, plus a certain percentage of delay or fatigue time points to predict work hours and prospectively arrange manpower [6]. In Singapore, scholars developed a nursing intensity system based on nursing diagnosis, which identifies the type of nursing diagnosis based on the type of intervention performed on the patient, quantifies the relationship between the nursing time required and the type of nursing diagnosis through a regression equation, and obtains the number of patients with different nursing diagnoses to predict the nursing work hours required per shift [7]. In addition, Japan has developed a Nursing Care Assignment Management (NCAM) system to visualise daily workloads and use the quantified workload data to efficiently assign staff and tasks [8]. In China, scholars have used the Hospital Information System (HIS) to obtain and predict workload data. In [9], the HIS system was combined with the work hour measurement method, and the nursing work hours of each operation were correlated with the frequency of HIS medical orders to establish an HIS-based nursing work our database. The relationship between the number of nursing hours and the number of nurses was calculated by linear regression analysis; for example, Nephrology: number of nurses = 1.577 + 0.030*x* working hours, predicted nursing manpower required for tomorrow = 1.577 + 0.030*x* total nursing hours of yesterday ÷ actual number of admissions yesterday ÷ expected number of admissions tomorrow. By programming the formula into the information system, the manager only needs to enter the number of hospital admissions for tomorrow to predict the number of people who will work on tomorrow.

Nursing projects are mostly derived from doctors' orders and nursing workflows and are selectively retrieved from information systems in a simple and operational way [10]. Overseas information systems mainly quantify the categorisation of patient need and use statistical analysis to identify the relationship between patient categories and workload, thereby predicting the number of nursing hours required for each category of patients and allocating manpower. Domestic information systems are based on the prediction of workload purely statistical nursing items to get nursing workload, but due to the imperfect data dictionary of information systems, in practice there are many nonchargeable items and indirect nursing work that are not included in the dictionary of medical advice, such as health education, linen change, material collection, and so on, which cannot be directly retrieved for statistics. In addition, the different information system environments of individual hospitals, the fact that standard nursing hours have not yet been unified, and the lack of uniform standards for screening nursing items make the results of some scholars' research not universally applicable and externally applicable [11, 12].

The contributions of this article are as follows:

This article proposes to build a nursing education management model based on small data, which provides ideas and references for nursing education management to effectively predict the evolution of student behavior and improve the level of precise service.

Based on the in-depth analysis of the concept and characteristics of small data, we design a dynamic portrait label system from two aspects of student surface behavior and deep driving factors and propose a research method to realize dynamic portrait and form an overall framework model.

The experimental group adopts the whole-course dual-mentor responsibility system holistic nursing practice model and comprehensively evaluates the two groups of nursing students, investigating the professional identity of the nursing students, the evaluation of the internship model, and the satisfaction of patient care services.

## 2. Related Work

### 2.1. A Predictor-Based Approach to Nursing Workload Prediction

Predictor-based methods for forecasting nursing workload are based on statistical methods such as regression analysis, which explores the factors inherent in nursing workload data, use the main influencing factors as predictors, and identify parameters to be determined in order to construct a mathematical model, followed by regression modelling of the identified predictors using polynomial Logistic, LASSO, and other methods [13]. Common predictors used overseas include Hours Per Patients Day (HPPD) [14], Case Mix Index (CMI) [12, 14], and International Classification of Function (ICF) [15].

In addition, studies have also shown that the Unit Activity Index (UAI), the number of discharges, transfers, or discharging (Admitting, Transferring, or Discharging Patients (ADT)) [16] have a positive impact on the inclusion of workload prediction. Reference [17] used multiple stepwise linear regression analysis to identify the factors influencing work hours and used them as classification factors to build a query table of direct nursing hours and indirect nursing hours required by different categories of patients. The table was used to identify the factors influencing the working hours and use them to establish a table of direct nursing hours and indirect nursing hours required by different categories of patients for dynamic manpower deployment. In [18], the results of a survey in a general adult ward in a tertiary care hospital showed that there was a linear relationship between the Barthel Index and Simple Clinical Score (SCS) of patients and the 24-hour direct nursing hours of patients, and a mathematical model using least squares and multiple linear regression was developed to identify the nursing hours of patients in different levels of care, thus enabling prediction of nursing workload.

### 2.2. Grey Dynamic Model

Grey systems theory consists of the use of dynamic predictive models to process incomplete small sample “information-poor” data to establish a quantitative relationship between the present and the future on a timeline, through which things can be predicted [19]. Nursing workload is the result of a combination of internal and external environmental factors, such as patients, environment, and nurses, which are covered by grey information. In contrast, workload measures are specific and defined, covered by white information, and meet the requirements for predictive modelling of data grey systems [20]. Reference [21] used a grey system prediction model to quantitatively predict trends in nurse performance, using 12 nurses in the unit as the study population and selecting four months of performance for each nurse as the basic data for modelling and predicting nurse performance over the next five months. Nursing performance is a quantitative assessment of nurses' workload, and the prediction of performance can indirectly help managers predict workload, scientifically set work standards, and reasonably assign work tasks.

### 2.3. Time Series Models

Time series models are forecasting methods that apply mathematical models to describe the changes in variables over time and their development patterns, that is, predicting their future values from the past and present values of the time series. The Autoregressive Integrated Moving Average Model (ARIMA) is one of the most widely used and well-known time series forecasting methods, which is able to integrate seasonal, trend, and random disturbance factors and has good forecasting function, and is gradually used in the field of health care [2]. Reference [22] used the ARIMA model to predict daily patient occupancy in a short-term acute care ward for optimal management of human resources, and the results showed that the model predicted with high accuracy for up to one week, which is a good guide for managers. Reference [23] used an ARIMA model prediction combined with linear correlation and regression analysis to obtain a predictive model for the number of obstetric outpatient card builds and deliveries. Reference [24] used time series analysis to construct a time series prediction model for daily nursing workload in the neurology department.

### 2.4. Discrete Event System Simulation

Discrete event simulation (DES) is a method of experimenting with computer simulation of discrete events for evaluating, predicting, and optimizing the efficiency of existing systems. The DES model was used by [25] to predict caregiver demand in a nursing home. Data on the daily care needs (ADLs) of older people were collected and entered into the DES model to predict the relationship between care workload and staffing to meet the daily care needs of older people so as to determine the optimal staffing for different care needs. Reference [26] collected 1 month of inpatient care data from a general hospital in Canada into the system and logically simulated the impact of different nurse-patient ratios on quality of care as well as nursing workload to help managers predict and optimally manage nursing workload. Discrete event simulation is a nurse-centred approach to workload prediction.

## 3. Questions to Ask

### 3.1. Small Data

The concept of small data was first discovered by Professor D. Eestrin of Cornell University, who believed that students' health could be dynamically monitored through the tracking of a full range of data on their daily behaviour [27]. There is no clear definition of small data, but it is accepted that small data is a collection of all data on a person or team-centred, multilevel behavioural pattern, and situational awareness. Over time, these data collections have become richer and richer, providing a powerful support for dynamic mining of student needs and preferences and behaviour patterns. Current research on small data focuses on personalised recommendations and accurate services, interest discovery and prediction, and theoretical discussions on the fusion of small data, but there is a lack of conceptual concepts for building academic app portraits based on small data.

### 3.2. Nursing Education Management Student Portrait

Traditional student portraits only capture data at a certain point in time; that is, they abstract a static model of a student's reality and history based on their behavioural characteristics, habits, and other data tags. A dynamic portrait of a student in nursing education management is one that introduces a time segment based on a conceptual picture of the student and uses a scientific approach to dynamically and continuously outline trends in the behavioural trajectories of students as they interact with the platform.

## 4. Overall Design

**Figure 1:**
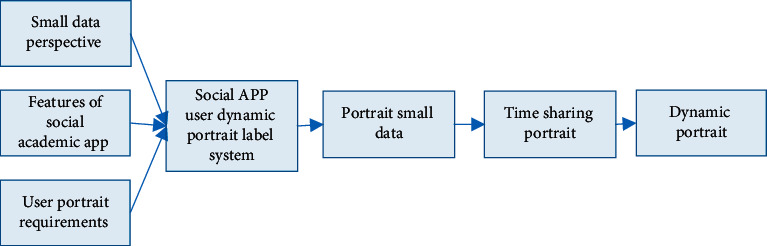
The process of constructing a student portrait for nursing education management based on small data.

The dynamic portrait model based on small data proposed in this paper places more emphasis on the relevance and three-dimensionality of the labelling system. Therefore, the process of the small data-based student profiling model for nursing education management should be divided into four segments, as shown in Figure 1:

 
*Step 1*. Design a three-dimensional dimensional label that includes deeper drivers of student behaviour from a small data perspective, combined with the characteristics of nursing education management.
 
*Step 2*. Collect and preprocess student data-based on the dimension labels.
 
*Step 3*. Classify the data according to the temporal information in the student microdata and use clustering algorithm to cluster the data in specific time segments to classify students into different groups and build a time-phased portrait, which will be stored in the database.
 
*Step 4*. Based on the time-phased portrait, identify the cluster centres of each time period (the cluster centres can be regarded as the typical representatives of the group of students).

The above design process not only refers to the basic aspects of traditional student profiling, but also incorporates the special features and requirements of the small data based dynamic profiling proposed in this paper, which ensures practical value and innovation.

## 5. Nursing Education Management Systems and Small Data Acquisition

### 5.1. Label Dimension

The psychological activity of students is an intrinsic factor that underpins behaviour, and any behaviour is driven by certain intentions before it occurs. Social psychologist Levin developed the field dynamics theory to analyse the driving forces and behavioural change processes that underpin individual behaviour. Field dynamics theory consists of field theory and dynamics theory, in which field theory defines “field” as the overall form of interdependence between the individual and the environment, also known as individual life space (LS). The individual's psychological and behavioural processes always take place and move within this space, which can be expressed in a functional equation as

(1) $$\begin{array}{c} B=f\left(P^{*}E\right)=f\left(LS\right), \end{array}$$

where *B* represents the externalised behavioural expression. *P* represents the intrinsic needs of the individual, *E* represents the psychological environment, that is, the situation that stimulates the intrinsic needs of the individual, and *f* is a function of the interaction between the individual and the environment. Thus, field theory considers individual behaviour as a result of the interaction between the subject and the situation.

### 5.2. Small Data Collection

**Figure 2:**
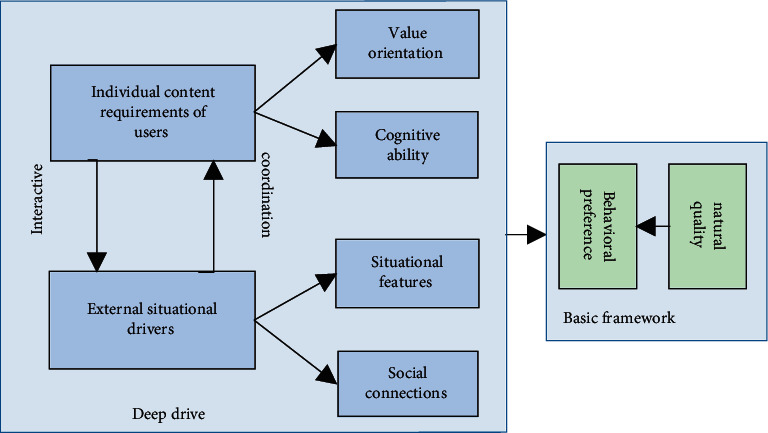
System of nursing education management student portrait dimensions.

Based on the field dynamics theory and the characteristics of nursing education management, this paper argues that the factors driving student behaviour in nursing education management include students' value orientations, cognitive abilities, situational characteristics, and social relationships. Combining the two basic factors of student natural attributes and behavioural preferences, a six-dimensional portrait system is constructed, as shown in Figure 2. The internal relationship of each dimension is as follows: students' natural attributes and behavioural preferences are the basic framework of the portrait, while values, cognitive abilities, situational characteristics, and social relationships drive the production of behaviour, among which values and cognitive abilities are driven by students themselves, that is, internal drive *P*, and situational and social situations are the stimulus of external situations (i.e., trigger). E. Behavioural preferences are the external effect of the combination of natural attributes, values, cognitive abilities, situational characteristics and social relationships. E. Behavioural preferences are the externalisation of a combination of natural attributes, values, cognitive abilities, situational characteristics, and social relationships.

Basic student information and care data are stored in the administration backend; behavioural preferences can be obtained from behavioural data stored in student logs and student-generated content; value orientations can be obtained by mining student-generated content or through research methods such as questionnaires and interviews (e.g., targeted electronic questionnaires distributed by the platform to understand students' visions and current needs); cognitive abilities can be obtained from authentication data submitted by students and analysis of interaction data. Cognitive skills can be obtained from student submissions and interaction data analysis; situational awareness can be obtained from sensors, location systems, and smart wearable devices; social relationships can be obtained from log mining and social network analysis.

### 5.3. Small Data Processing

**Figure 3:**
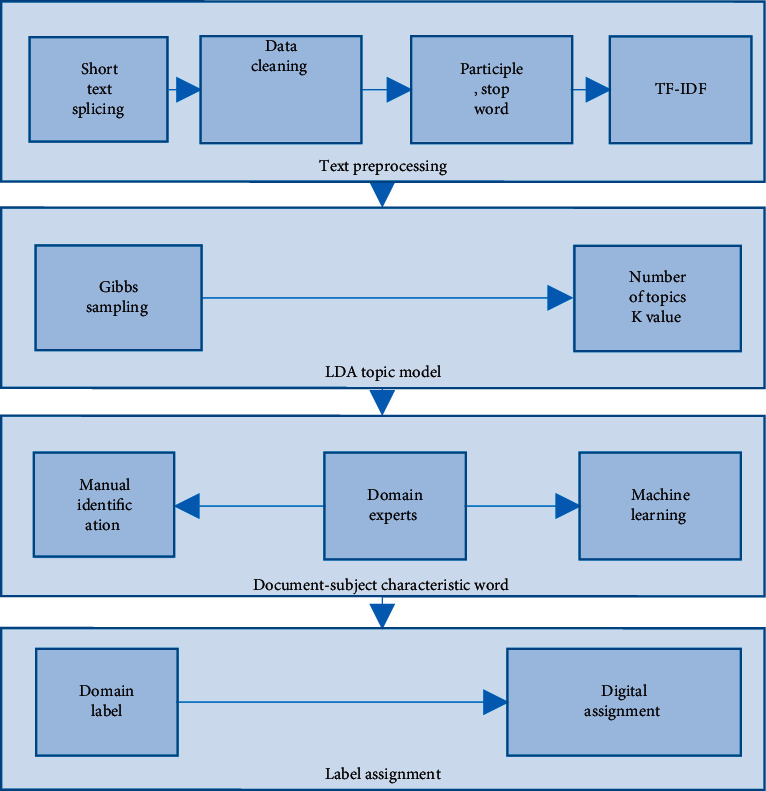
Steps for modelling unstructured text based on LDA topic models.

In the past, most of the data used in student profiles were basic attribute data and behavioural data, which could be coded and transformed numerically or simply processed for direct experimental analysis. This paper builds on this foundation by incorporating comment or posting text (i.e., content features). The professional and domain-specific nature of nursing education management and the fact that students' nursing topics are often related to their own specific domains makes it possible to mine and analyse students' areas of interest from the textual content. Therefore, this paper proposes a text data modelling approach based on the LDA topic model (see Figure 3):

*Step 1*. The text content was collected, stitched, and stored as text documents by student IDs, cleaned and imported into the domain word list for Chinese word separation and deactivation filtering, and the original text was cut into a sequence of feature words; then, the TF-IDF was used to calculate feature word weights from both word frequency and importance perspectives, and important feature words were retained.
*Step 2*. Use the easy-to-use Gibbs sampling in the LDA (Latent Dirichlet Allocation) topic model to mine the implied topics of the text; that is, project the complex text into the potential topic space according to the statistical idea, and obtain the “document-topic” distribution *θ* topic distribution *θ* and “topic-featured word” distribution:
  (2) $$\begin{array}{c} \text{perplexity}\left(D\right)=\exp\left[-\frac{\sum_{d=1}^{M}\log\left(P{\left(W\right)}_{d}\right)}{\sum_{d=1}^{U}N_{d}}\right],\\ p\left(w\right)=p\left(z|d\right)*p\left(w|z\right). \end{array}$$
*Step 3*. After obtaining the “document-topic-feature word” matrix, if the sample is small, each topic word cluster can be identified manually, taking into account the distribution of words and semantic relationships under the topic. As most of the nursing education management apps are industry vertical platforms, such as the Dingxiangyuan App for the medical field and the House of Economics and Management App for the management field, we can consult with experts in the relevant fields to set a domain label for each document that summarises its characteristics when identifying thematic feature words.
*Step 4*. After obtaining the domain labels for each document, the labels are digitally assigned, for example, the labels “internal medicine,” “surgery,” and “Chinese medicine” are assigned {1, 2, 3}, respectively. The result is the identification of student interest areas from student-generated content and their conversion into numerical values.

The above method can quantify the textual data and also reveal the textual characteristics at the thematic level, which is both practical and scientific, and achieves the research goal of combining textual data and other data to portray the portrait, avoiding distortion of the portrait to the greatest extent possible.

## 6. Case Studies

### 6.1. Study Subjects

A convenience sampling method was used to include 200 nursing students who underwent a clinical internship in a tertiary care hospital from August 2014 to April 2015. The experimental group consisted of 200 nursing students who underwent clinical internship from August 2014 to April 2015, and the control group consisted of 200 nursing students who underwent an internship in the same hospital, all with bachelor's degree. During the study period, 3 nurses were dropped out of the experimental group and 6 from the control group. The reason for the dropout was that the nursing students were transferred to other hospitals at the end of their internship and were unable to complete the comprehensive assessment and related questionnaires. Therefore, a total of 192 nursing students in the experimental group and 185 in the control group were included in this study [28].

### 6.2. Evaluation Methods

At the end of the internship, nursing students are assessed in a unified manner, including theoretical and operational assessments, with a score of 100 points each. Each student is evaluated by the first- and second-level managers according to the “Internship Performance Assessment Form,” which covers five major categories: professionalism, professional skills, communication skills, study and research skills, and teamwork, each with 20 points, totalling 100 points [29].

A survey of nursing students at the end of their placement was conducted using the “Nurse Professional Identity Scale” developed by the Department of Nursing Management, Faculty of Medicine, University of Tokyo, Japan. The scale consists of 21 items with 7 dimensions: a sense of control, a sense of consistency, a sense of meaningfulness, sense of self-efficacy, sense of self-determination, sense of organisational influence, and sense of patient influence. The items are rated on a 7-point Likert scale, according to “not at all,” “less,” “less,” “not sure,” “not sure,” “not at all,” and “not sure.” The dimensional score is the average of the scores of the included items. The higher the score, the higher the degree of professional identity of nursing students. Cronbach's alpha coefficient for the Chinese version of the scale was 0.84 and the content validity was 0.92. In this study, Cronbach's alpha coefficient was 0.817.

A self-made patient care service satisfaction questionnaire was distributed to patients at the end of the practice, including five dimensions of nursing student service quality, professional skills, patient education, humanistic care, and overall evaluation, with a total of 16 items. “Dissatisfied,” “average,” “satisfied,” and “very satisfied” were assigned a score of 1 to 5, respectively. The higher the score, the higher the patient's satisfaction with the nursing student. Six experts in nursing education, nursing management, and clinical nursing were invited to evaluate the relevance of each item to the content expressed in the questionnaire on a 4-point scale (i.e., 1 = not relevant, 2 = weakly relevant, 3 = strongly relevant, and 4 = very relevant). The Cronbach's alpha coefficient for this questionnaire was 0.923.

A self-made questionnaire was used to survey nursing students at the end of the placement, which included five items: rationality, replicability, the quality of teaching, quality of management, and overall evaluation of the placement model.

### 6.3. Results

Comparison of overall assessment scores of nursing students is presented in Table 1.

Clinical practice is an integral part of higher nursing education, and the quality of clinical teaching is crucial to the overall standard of higher education. It is an innovation and development of the traditional mobility practice paradigm, adhering to the student-centred talent training concept, based on the development of nursing students, based on clinical nursing practice, facing the frontier of discipline development, adapting to the educational market demand, aiming to cultivate excellent nursing students, and devoting to the comprehensive training of nursing students' knowledge, ability, and quality so as to become talents with greater development potential. As shown in Table 1, the experimental group was the first to be trained in nursing. As shown in Table 1, the assessment scores and overall practice performance of nursing students in the experimental group were higher than those of the control group (*P* < 0.05), and the comparison was statistically significant in terms of professional quality, professional skills, communication ability, study and research ability, and team writing (*P* < 0.05) [30].

Comparison of professional identity of nursing students is shown in Table 2.

Professional identity is a socialised self-identification of the individual's values, attitudes, behaviour, knowledge, and skills in their professional role, and is the process of integration and development of self-value and professional value that overcomes the sense of professional externalities and alienation. Clinical practice is a critical period for nursing students to strengthen their professional awareness, experience their professional values, and transform their social roles and has an important impact on the formation and development of their professional identity. As shown in Table 2, through the clinical practice of the whole nursing internship model of double censorship, in which the mentor is responsible for the whole process and the nursing students are independently assigned to beds at the end of the internship, the nursing students in the experimental group have a higher sense of grasp and consistency of the professional role of nurses than those in the control group (*P* < 0.05), their clinical skills have been improved, their degree of nursing decision-making and influence on patients has been deepened, and their sense of self-worth has been elevated, and they are higher than those in the control group in the above related dimensions (*P* < 0.05). All comparisons were higher than those of the control group (*P* < 0.05). It is possible that this is due to the limited time spent on rotation in different departments and limited participation in the nursing organisation. The nursing students in both groups scored lower on this dimension, with there was no statistical difference between the two groups (*P* > 0.05). In conclusion, the holistic nursing practice model with dual membership can continuously enhance the professional experience of nursing students and promote the positive transition of nursing students to their social roles [30].

Comparison of patient satisfaction with care services is shown in Table 3.

Table 3 shows that the overall satisfaction of patients with nursing students in the experimental group was higher than that of the control group (*P* < 0.05), and there was a statistical difference in the comparison of satisfaction in terms of service quality, professional skills, patient education, and humanistic care (*P* < 0.05). Patient satisfaction is the evaluation yardstick of quality nursing service and is the starting point and footing point of nursing practice. The nursing students are the new force in the nursing team and should inherit and carry forward the “patient-centred” service concept. Under the dual-tutor system, the tutors focus on the cultivation of nursing students' service consciousness and professional ability, supervising them to implement basic nursing care and health education for patients and guiding them to communicate effectively with patients; the nursing department organises regular humanities lectures and health education competitions to motivate nursing students to provide quality nursing services [31].

Comparison of nursing students' evaluation of placement models is shown in Table 4.

The results of the nursing students' evaluation of the practice model (Table 4) show that the overall nursing practice model with dual tutors is more reasonable than the traditional practice model and is highly scalable. In the construction of this model, a complete three-level supervision system, a dual-tutor teaching system, a PDCA accountability system for continuous improvement, and a multichannel teacher-student interaction platform have been formed after continuous experience summing up and perfecting practice, which is worth promoting at one time.

## 7. Experimental Analysis

**Figure 4:**
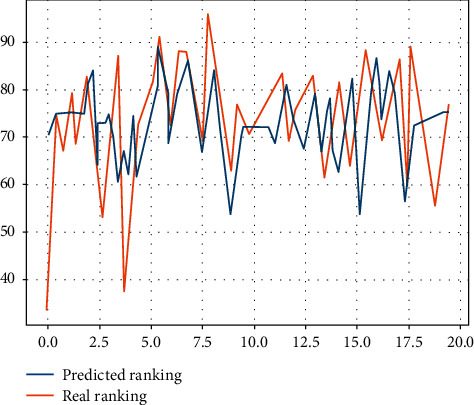
Predicted performance for different management.

In the process of data collection and analysis, the metadata that is exchanged as information is standardised in order to fully exploit, share, and utilise information resources. The standardisation of nursing language has been researched earlier overseas, and information systems predict nursing workload based on the Nursing Outcome Classification (NOC), the North American Nursing Diagnostic Association Nursing Diagnosis (NANDA), and other standardised language captures, providing a good basis for full data mining and analysis. As nursing work records in China are still dominated by semistructured, nonstandardised data, the lack of a common standardised nursing language and terminology system has resulted in a large amount of nursing workload data that cannot be directly analysed and processed, and a low rate of sharing and utilisation of data resources across institutions and systems as shown in Figure 4, with different management of prediction models.

**Figure 5:**
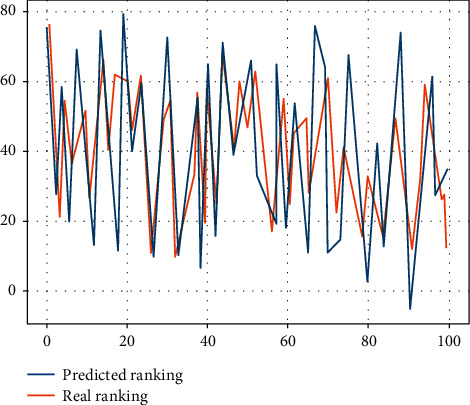
Different prediction levels.

In the context of the era of medical informatization, it is urgent for China to establish a unified coordination mechanism, promoted by the state and professional academic organizations, to develop a standardised system of nursing data suitable for China, such as the different prediction modules shown in Figure 5.

**Figure 6:**
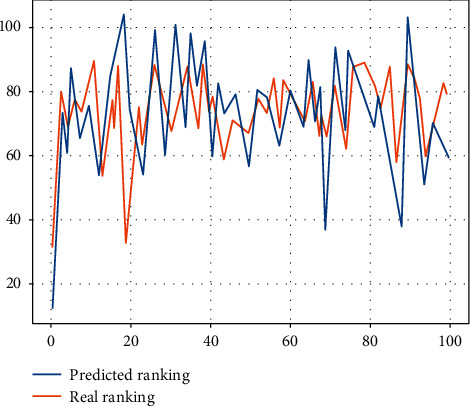
Short-term effects of care.

As shown in Figure 6 for the short-term nursing effect, the difficulty in achieving the prediction of nursing workload is not in the use of data mining tools, but in choosing which data mining tools are more applicable to the reality of nursing workload in China, which currently presents diverse, complex, and fragmented data. When predicting, the traditional regression approach inevitably leads to errors and even biases, and due to the expanding volume of data, there is an urgent need for working methods that are more in line with the volume of nursing data. Currently, methods such as support vector machines and random forests are increasingly becoming mainstream mining tools, and more suitable research methods should be actively incorporated in the prediction methods of nursing workload.

## 8. Conclusions

This paper proposes the construction of a nursing education management model based on small data to provide ideas and references for nursing education management to effectively predict the evolutionary trends of student behaviour and improve the level of precise services. The experimental group adopted a full dual-mentor responsibility system holistic nursing practice model, specifically including a complete three-level supervision system, dual-mentor teaching system, PDCA responsibility system holistic nursing practice pathway for continuous improvement, and a multichannel teacher-student interaction platform.

## Figures and Tables

**Table 1:** Comparison of the comprehensive assessment scores of nursing students in the two groups $\left(\bar{x}\pm s\right)$.

Project	Experimental group (*n* = 182)	Control group (*n* = 165)	*t*/*t*′ value	*P* value
Theoretical assessment	79.70 ± 8.12	77.19 ± 6.78	3.108	0.002
Operation assessment	85.48 ± 7.33	82.56 ± 8.25	3.491	<0.001
Practice performance	72.02 ± 10.12	66.58 ± 12.27	4.472	<0.001
Professional quality	14.72 ± 1.92	13.55 ± 1.94	5.621	<0.001
Professional skills	15.04 ± 2.22	14.29 ± 2.98	2.631	<0.001
Communication skills	13.25 ± 2.21	11.48 ± 2.45	5.843	0.009
Learning and scientific research ability	14.81 ± 2.38	13.57 ± 5.24	4.512	<0.001
Teamwork	14.21 ± 2.1	13.26 ± 2.49	3.815	<0.001

**Table 2:** Comparison of professional identity between the two groups of nursing students $\left(\bar{x}\pm s\right)$.

Project	Experimental group (*n* = 182)	Control group (*n* = 165)	*t* value	*P* value
Sense of grasp	5.86 ± 0.99	5.33 ± 1.04	4.862	<0.001
Sense of consistency	5.52 ± 1.41	4.36 ± 1.68	6.988	<0.001
Sense of meaning	5.88 ± 1.25	5.40 ± 1.36	3.426	<0.001
Self-efficacy	5.91 ± 0.85	5.64 ± 0.93	2.826	0.005
Self-determination	4.35 ± 1.84	3.76 ± 1.92	2.922	0.004
Sense of organisational influence	3.66 ± 1.53	3.38 ± 1.65	1.640	0.102
Patient's sense of influence	5.15 ± 1.20	4.39 ± 1.33	5.465	<0.001

**Table 3:** Patient care service satisfaction $\left(\bar{x}\pm s\right)$.

Project	Experimental group (*n* = 182)	Control group (*n* = 165)	*t* value	*P* value
Service quality	3.91 ± 0.84	3.62 ± 0.93	3.056	0.002
Professional skills	4.00 ± 0.88	3.46 ± 0.95	5.496	<0.001
Patient education	4.05 ± 0.92	3.53 ± 0.86	5.425	<0.001
Humanistic concern	3.60 ± 0.89	3.26 ± 0.87	3.592	<0.001
Overall evaluation	3.86 ± 0.94	3.52 ± 0.97	3.314	0.001

**Table 4:** Evaluation of placement patterns by nursing students in both groups $\left(\bar{x}\pm s\right)$.

Project	Experimental group (*n* = 182)	Control group (*n* = 165)	*t*/*t*′ value	*P* value
Rationality	4.26 ± 0.76	3.70 ± 0.94	6.075	<0.001
Generalizability	4.40 ± 0.73	3.34 ± 0.82	12.760	<0.001
Teaching quality	4.20 ± 0.78	3.62 ± 1.02	5.900	<0.001
Management quality	4.25 ± 0.79	3.55 ± 0.95	7.382	<0.001
Comprehensive evaluation	1.30 ± 0.81	3.53 ± 0.89	8.424	<0.001

## Data Availability

The data underlying the results presented in the study are included within the manuscript.

## References

[1] Han R., Mo W., Shao D. (2016). Research on the construction strategy of information model for manchuria style architecture and its application. *International Journal of Smart Home*.

[2] Liu Y., Li D. Research on construction of nursing knowledge portal based on big data.

[3] Fleming S. E., Boyd A., Ballejos M. (2013). Goal setting with type 2 d. *The Diabetes Educator*.

[4] Hu J., Zhang M. (2015). Research on the construction of liberal arts graduate student learning situation-A Case study of the tourism management major in guangdong province. *Higher Education Studies*.

[5] Randall E. J. (2010). Research and its application in the advanced nursing series. *Journal of Advanced Nursing*.

[6] Appelbaum P. S., Anatchkova M. D., Albert K., Dunn L. B., Lidz C. W. (2012). Therapeutic misconception in research subjects: development and validation of a measure. *Clinical Trials*.

[7] Vanneste D., Vermeulen B., Declercq A. (2013). Healthcare professionals’ acceptance of BelRAI, a web-based system enabling person-centred recording and data sharing across care settings with interRAI instruments: a UTAUT analysis. *BMC Medical Informatics and Decision Making*.

[8] Frick J. R., Goebel J., Schechtman E., Wagner G. G., Yitzhaki S. (2004). Using analysis of gini (ANoGi) for detecting whether two sub-samples represent the same universe: the SOEP experience. *IZA Discussion Papers*.

[9] Bahn S., Barratt-Pugh L. (2013). Getting reticent young male participants to talk: using artefact-mediated interviews to promote discursive interaction. *Qualitative Social Work*.

[10] Levin L., Peled E. (2011). The attitudes toward prostitutes and prostitution scale: a new tool for measuring public attitudes toward prostitutes and prostitution. *Research on Social Work Practice*.

[11] Wall M. L., Carraro T. E. (2009). Kuhn’s revolutionary theory and its influence on the construction of nursing knowledge. *Revista Latino-Americana de Enfermagem*.

[12] Intree C., Srisa-Ard B., Sirisuth C. (2008). The development of a knowledge management (KM) model for the faculty of nursing, ratchathani university. *Education Journal of Thailand*.

[13] Clark C., Farnsworth J., Landrum E. (2009). Development and description of the incivility in nursing education (INE) survey. *Journal of Theory Construction & Testing*.

[14] Helmig B., Ingerfurth S., Pinz A. (2014). Success and fn organizations: theoretical foundations, empirical evidence, and future research. *Voluntas: International Journal of Voluntary and Nonprofit Organizations*.

[15] Weisberg R. H., Zheng L. (2008). Hurricane storm surge simulations comparing three-dimensional with two-dimensional formulations based on an Ivan-like storm over the Tampa Bay, Florida region. *Journal of Geophysical Research: Oceans*.

[16] Marzieh H. Z., Reza A. M., Suleiman A., Gholizadeh R. H., Karimi-Moonaghi H. (2015). Knowledge creation in nursing education. *Global Journal of Health Science*.

[17] Zhang L., You X., Jiao J., Helo P. (2009). Supply chain configuration with co-ordinated product, process and logistics decisions: an approach based on Petri nets. *International Journal of Production Research*.

[18] Wojna A. (2003). Center-based indexing in vector and metric spaces. *Fundamenta Informaticae*.

[19] Marchman V. A., Fernald A. (2010). Speed of word recognition and vocabulary knowledge in infancy predict cognitive and language outcomes in later childhood. *Developmental Science*.

[20] Vanderven K. (2011). The road to intergenerational theory is under construction: a continuing story. *Journal of Intergenerational Relationships*.

[21] Giltrap D. L., Saggar S., Singh J. S., Harvey M. J., Mcmillan A. M. S., Laubach J. (2012). Field-scale verification of nitrous oxide emission reduction with DCD in dairy-grazed pasture using measurements and modelling. *Soil Research*.

[22] Harvey S. A., Wandersee J. R. (2010). Publication rate of abstracts of papers and posters presented at Medical Library Association annual meetings. *Journal of the Medical Library Association: JMLA*.

[23] Sider L. H., Heaton M. P., Chitko-McKown C. G. (2013). Small ruminant lentivirus genetic subgroups associate with sheep TMEM154 genotypes. *Veterinary Research*.

[24] Wallman A., Sporrong S. K., Gustavsson M., Lindblad A. K., Johansson M., Ring L. (2011). Swedish students’ and preceptors’ perceptions of what students learn in a six-month advanced pharmacy practice experience. *American Journal of Pharmaceutical Education*.

[25] Strijbos J., Engels N., Struyven K. (2015). Criteria and standards of generic competences at bachelor degree level: a review study. *Educational Research Review*.

[26] Kundakcı N., Ayşegül Tuş I. (2016). Integration of MACBETH and COPRAS methods to select air compressor for a textile company. *Decision Science Letters*.

[27] Gage-Bouchard E. A., Devine K. A. (2014). Examining parents’ assessments of objective and subjective social status in families of children with cancer. *PLoS One*.

[28] Blundell J., Scott S., Harris D., Huddlestone J., Richards D. (2020). Workload benefits of colour coded head-up flight symbology during high workload flight. *Displays*.

[29] Gao Z., Zhai G., Deng H., Yang X. (2020). Extended geometric models for stereoscopic 3D with vertical screen disparity. *Displays*.

[30] He S., Liang B., Tähkämö L., Maksimainen M., Halonen L. (2020). The influences of tunnel lighting environment on drivers’ peripheral visual performance during transient adaptation. *Displays*.

[31] Weech S., Kenny S., Calderon C. M., Barnett-Cowan M. (2020). Limits of subjective and objective vection for ultra-high frame rate visual displays. *Displays*.

